# Can endocan serve as a molecular “*hepatostat*” in liver regeneration?

**DOI:** 10.1186/s10020-023-00622-9

**Published:** 2023-02-27

**Authors:** Sinan Efe Yazici, Mustafa Emre Gedik, Can Berk Leblebici, Kemal Kosemehmetoglu, Gurcan Gunaydin, Ahmet Bulent Dogrul

**Affiliations:** 1 Department of General Surgery, Hacettepe University School of Medicine, Sihhiye, 06100 Ankara, Turkey; 2 Department of Basic Oncology, Hacettepe University Cancer Institute, Sihhiye, 06100 Ankara, Turkey; 3 Department of Pathology, Hacettepe University School of Medicine, Sihhiye, 06100 Ankara, Turkey

**Keywords:** Endocan, Liver transplantation, Hepatectomy, Regeneration, eEF2K

## Abstract

**Background:**

Intriguingly, liver regeneration after injury does not induce uncontrolled growth and the underlying mechanisms of such a “*hepatostat*” are still not clear. Endocan, a proteoglycan, was implicated in liver regeneration. It can support the function of hepatocyte growth factor/scatter factor in tissue repair after injury. Endostatin, a 20 kDa C-terminal fragment of collagen XVIII, may modulate the cessation of liver regeneration. eEF2K, a protein kinase that regulates protein synthesis, can regulate angiogenesis. Thus, we investigated the role of endocan, endostatin and eEF2K during normal liver regeneration.

**Methods:**

Serum samples and regenerating remnant liver tissues were obtained on various days after partial hepatectomy in rats. mRNA expression levels of *Vegf* and *Pcna* were analyzed in addition to immunohistochemical evaluations. Liver tissue protein levels of endostatin, endocan and p-eEF2K/eEF2K were determined with Western blot. Serum levels of endostatin and endocan were assessed with ELISA.

**Results:**

*Pcna* expression level in residual liver tissues peaked on day-1, while *Vegf* expression reached its highest level on days 1–3 after partial hepatectomy (70%). Endocan activity declined gradually on days 1–7. The decrease in liver endocan expression was accompanied by an increase in serum endocan levels. Partial hepatectomy induced a rapid increase in liver endostatin levels. Following its surge on day-1, endostatin expression gradually declined, which was accompanied by a peak in serum endostatin. Finally, partial hepatectomy was shown to regulate eEF2K; thus, increasing protein translation.

**Conclusions:**

We revealed possible mechanistic insights into liver regeneration by examining the associations of *Pcna*, *Vegf*, endocan, endostatin, eEF2K with hepatic regeneration after partial hepatectomy. Indeed, endocan might serve as a useful biomarker to monitor clinical prognosis in a plethora of conditions such as recovery of donor’s remaining liver after living-donor liver transplant. Whether endocan might represent a strategy to optimize liver regeneration when given therapeutically needs to be investigated in future studies.

## Introduction

**Fig. 1:**
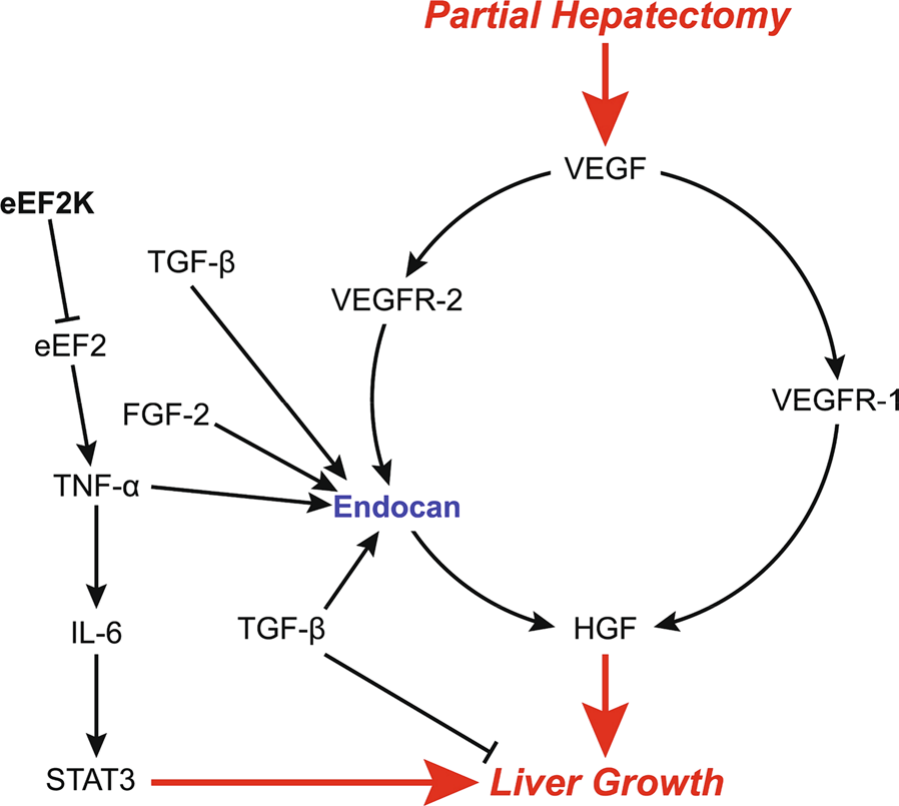
Factors implicated in liver regeneration after partial hepatectomy. Given the literature and the findings of the current study, we suggest that endocan may play a pivotal role in terms of liver regeneration after partial hepatectomy. The figure depicts the summary of critical findings from the literature as well as proposing a probable role for endocan in hepatic regeneration. We investigated several key molecules in the axis between partial hepatectomy and liver growth during regeneration

The liver bears an exceptional feature in terms of restoration of its anatomical volume and mass after toxic injury or surgical resection. It responds to such alterations with tissue regeneration (Black et al. 2004; Dar et al. 2019; Liu and Man 2021; Jimenez-Castro et al. 2019). The optimal mass that the liver should achieve after partial hepatectomy (PH) or transplantation depends on several parameters such as liver/body mass ratio and angiogenesis (Michalopoulos 2014). Hepatocyte regeneration and then angiogenesis occur after PH. The liver reaches to its optimal functional volume and mass between postoperative days 7 and 10 in rats (Dogrul et al. 2010). When the liver achieves its optimal functional size, the regeneration process ceases. Thus, the liver regeneration does not induce pathological angiogenesis or uncontrolled growth (Dogrul et al. 2010). Transforming growth factor-β (TGF- β) and activins were proposed to be implicated in this process (Fig. 1) (Zimmermann 2004). Although this phenomenon has long been known, the underlying mechanisms are not clear and studies are still looking for such a “hepatostat”, which would induce or inhibit the signals associated with growth or angiogenesis, resulting in the precise maintaining of the liver size (Michalopoulos 2010).

The liver demonstrates a critical regenerative capacity in which hepatocytes and biliary epithelial cells start proliferating after acute liver injury in order to restore the functions as well as the architecture of the liver parenchyma (Dogrul et al. 2010; Michalopoulos and DeFrances 1997; Michalopoulos 2007). The liver lobule is known to be organized into concentric zones where hepatocytes express varying metabolic enzymes, and different liver zones seem to contribute to hepatocyte homeostasis. In a recent seminal study, Wei et al. demonstrated that different regions of the liver lobule differ in terms of hepatocyte turnover, and zone 2 seems to be the primary source of new hepatocytes during regeneration (Wei et al. 2021). In another important study, He et al. reported zone 2 to show the highest proliferative activity and to contribute the most to liver regrowth (He et al. 2021). Indeed, the regeneration of liver bears complex responses and processes that involve the interaction of several types of cells such as hepatocytes, hepatic stellate cells, endothelial cells and inflammatory cells in a tightly coordinated manner (Campana et al. 2021). Signals responsible from such responses have long been investigated. Liver regeneration after 70%-PH in rodents has become a standard approach for investigating regenerative organ growth (Michalopoulos 2010). Genes associated with cell cycle control (*i.e.* stimulator and inhibitor genes) may prove to be crucial for precise control of cell growth by working in a balance after hepatectomy (Kountouras et al. 2001). Activation of several transcription factors has been implicated in the control of hepatic regeneration; however, the regulation of such transcription factors is poorly understood.

In light of the current literature, the liver seems to “know” when to start and stop growing (Kountouras et al. 2001). Even though this regeneration process has been histologically well demonstrated, molecules which orchestrate the liver regeneration have been only partially understood. Several growth factors, which were implicated in cell cycle control, have been investigated. Various pro- and anti-angiogenic factors have been well studied for many types of solid tumors; thus, a myriad of endogenous angiogenesis activators and inhibitors have been discovered.

One such molecule is endocan (endothelial cell‑specific molecule‑1 [ESM‑1]), which is indeed a potential immunoinflammatory marker that is related with cardiovascular disease as well as tumor angiogenesis (Matano et al. 2014; Chen et al. 2021; Balta et al. 2015). Endocan binds to lymphocyte function-associated antigen 1 (LFA-1) on human lymphocytes, monocytes, Jurkat cells; and inhibits its binding to intercellular adhesion molecule 1 (ICAM-1) and reducing LFA-1-mediated leukocyte activation as well as affecting the recruitment of circulating lymphocytes to inflammatory sites (Bechard et al. 2001a; Yahsi and Gunaydin 2022). Indeed, endocan was suggested to be a predictor of survival for patients with liver cirrhosis (Toshikuni et al. 2015) as well as a biomarker for steatosis (Erman et al. 2020; Klisic et al. 2020), which is known to be associated with defective liver regeneration (Allaire and Gilgenkrantz 2018). Endocan may regulate hepatocyte growth factor/scatter factor-mediated mitogenic activity (Bechard et al. 2001b). Zhao et al. recently reported that endocan played a role in the regeneration of residual liver after associating liver partition and portal vein ligation for staged hepatectomy (ALPPS), probably via the interaction with the hepatocyte growth factor/c-Met pathway (Zhao et al. 2020a). However, the exact role of endocan in liver regeneration is yet not clear.

Endostatin, which is a 20 kDa C-terminal fragment of collagen XVIII, is an inhibitor of angiogenesis produced by hemangioendothelioma (Dogrul et al. 2010; O’Reilly et al. 1997). It is one of the important endogenous factors that play an active role in tissue homeostasis. Endostatin was proposed to have a relationship with regeneration capacity after hepatectomy (Colakoglu et al. 2007). In addition, endostatin, which is produced by hepatocytes, was suggested to modulate the cessation of regeneration process by inhibiting angiogenesis (Dogrul et al. 2010). Systemic administration of endostatin inhibits angiogenesis and tumor growth as well as preventing growth of microscopic metastases (Colakoglu et al. 2007). Another molecule is eukaryotic elongation factor 2 kinase (eEF2K), which is a highly conserved protein kinase that regulates protein synthesis. eEF2K phosphorylates eukaryotic elongation factor 2 (eEF2), which is an essential factor for protein synthesis, and thus inhibits the eEF2 function (Ryazanov and Spirin 1990). eEF2k phosphorylates and inactivates eEF2, resulting in the inhibition of peptide-chain elongation. It can be activated by stimuli which are generally increased in stress- or starvation-related conditions (*e.g.*, by AMP-activated protein kinase) (Johanns et al. 2017; Gunaydin and Gedik 2019). eEF2K may promote cell proliferation, survival and tumorigenesis as well as regulating the cell cycle, autophagy, apoptosis and angiogenesis (Zhang et al. 2021). eEF2K is highly expressed in the liver tissue (Palasca et al. 2018). It has a crucial regulatory role in cell proliferation. Furthermore, it is implicated in liver diseases such as liver cancer and hepatitis (Gonzalez-Teran et al. 2013; Ballard et al. 2021). As such, eEF2K may be a promising therapeutic target in such diseases (Liu and Proud 2016). To the best of our knowledge, no study in the literature has investigated the role of endocan or eEF2K during normal liver regeneration. Therefore, we aimed to evaluate the role of endocan, a proteoglycan, and eEF2K in terms of liver regeneration and angiogenesis after PH in rats.

## Methods

### Experimental design

In order to assess the association of endocan with various other molecules implicated in liver regeneration and angiogenesis, hepatectomies were performed in rats. Normal, efficient regeneration was induced by 70% hepatectomy via a standard approach commonly used for investigating regenerative organ growth (Michalopoulos 2010). In order to reveal an association with pro-regenerative, pro-angiogenic and anti-angiogenic signals with endocan, proliferating cell nuclear antigen (PCNA), a universal marker of proliferating cells, vascular endothelial growth factor (VEGF) and endostatin as well as eEF2K were analyzed in a temporal manner.

### Animals

All laboratory animals used for the experiments were obtained, maintained and handled in accordance with the laws and institutional guidelines, as approved by the institutional review board of Hacettepe University (Approval number: 2020/05-01). Male Wistar albino rats aged 7 weeks (weighing between 300 and 350 g) were used and kept on a 12 h day/night cycle with ad libitum access to food and water.

### Animal surgery

The animals were fasted for 8 h before hepatectomy. A mixture of 5 mg/kg xylazine (Rompun Bayer, Turkey) and 30 mg/kg ketamine hydrochloride (Ketalar Panker Davis, Turkey) was administered *i.p.* for anesthesia/analgesia. 70% hepatectomy was performed on the animals in accordance with the technique described by Higgins and Anderson (Higgins and Anderson 1931; Nevzorova et al. 2015). The rats were randomized into 8 groups (n ≥ 3), which were defined as day-0, -1, -2, -3, -5, -7, -10,-14; denoting the days that the rats were sacrificed after PH. Serum samples were collected and regenerating remnant livers were removed on the day of sacrification. A portion of each liver specimen was fixed in 10% formalin solution for immunohistochemistry; a portion of the liver specimen was dissected in RIPA lysis buffer (Thermo Fisher Scientific Inc., Waltham, Massachusetts, USA) for protein analyses and a portion of the liver specimen was kept in RNA*later* stabilization solution (Thermo Fisher Scientific Inc., Waltham, Massachusetts, USA) for RT-qPCR analyses. mRNA expression levels of *Vegf* and *Pcna* were analyzed in addition to immunohistochemical evaluations. Liver tissue protein levels of endostatin, endocan and p-eEF2K/eEF2K were determined with Western blot and serum levels of endostatin and endocan were assessed with ELISA.

### Immunohistochemistry

Regenerative and angiogenetic activities of liver tissues were evaluated by immunohistochemical assessments of PCNA and VEGF. PCNA and VEGF stainings were performed on 4 μm thick slices obtained from formalin-fixed, paraffin-embedded liver tissues using an automated system (Leica BondMax II) via standard staining procedures. Following deparaffinization and antigen recovery by boiling in citrate buffer (pH: 6), the slices were stained with the primary antibodies (NeoMarkers; [monoclonal PCNA, Cat. # MS-106-P, 1/1000 concentration] and [VEGF Ab-7, Cat. # RB-222-R7, 1/100 concentration], EDTA solution, 10 min, Labvision Corporation, Fremont, California, USA) according to the manufacturer’s instructions. The slices were then incubated with biotinylated goat anti-mouse antibodies followed by streptavidin conjugated to horseradish peroxidase (Polyvalent Ultra-Tek Lab Pack Kit; ScyTek Laboratories Inc., Logan, UT, USA). Diaminobenzidine was used as the chromogen (DakoCytomation, Glostrup, Denmark).

Nuclei stained brown were considered as positive for PCNA. The PCNA labeling index was calculated as the proportion of PCNA-positive hepatocytes per 1000 hepatocytes counted under a light microscope at 40X magnification by two pathologists (blinded evaluation).

VEGF immunohistochemical staining results were also evaluated by two pathologists via a semiquantitative method according to the degree of VEGF positive cells (blinded evaluation) (Dogrul et al. 2010). A four-tiered scoring system on a scale ranging from 0 to 3 was used for VEGF expression (0: no staining, 1: VEGF expression only in periportal hepatocytes, 2: a multilayered VEGF expression starting from periportal to perisinusoidal hepatocytes sparing pericentral hepatocytes, 3: complete bridging VEGF expression in periportal, perisinusoidal, and pericentral hepatocytes) (Fig. 4a–h).

### Western blot

Freshly resected liver tissue samples were lysed in RIPA lysis and extraction buffer (Cat. # 89900, Thermo Fisher Scientific, Waltham, MA, USA) supplemented with Halt protease and phosphatase inhibitor cocktail (Cat. # 78442, Thermo Fisher Scientific, Waltham, MA, USA) for 30 min. Protein quantitation was carried out by Pierce BCA Protein Assay Kit (Cat. # 23225, Thermo Fisher Scientific, Waltham, MA, USA). Endocan (Anti-ESM1 antibody, Cat. # ab103590, Abcam Plc, Cambridge, UK), endostatin (COL18A1 Antibody, Cat. # sc-32720, Santa Cruz Biotechnology, Dallas, Texas, USA), eEF2K (Cat. # 3692S, Cell Signaling Technology Inc., Danvers, Massachusetts, USA) and phospho-eEF2K (Cat. # 3691S, Cell Signaling Technology Inc., Danvers, Massachusetts, USA) protein expression levels were investigated in remnant liver tissues obtained from the animals that were sacrificed 0, 1, 2, 3, 5, 7, 10, 14 days after PH via Western blot. β-actin (Cat. # 4970s, Cell Signaling Technology Inc., Danvers, Massachusetts, USA) was used as the Western blot loading control. Anti-rabbit IgG, HRP-linked Antibody (Cat. # 7074S, Cell Signaling Technology Inc., Danvers, Massachusetts, USA) was used as HRP (Horseradish Peroxidase) conjugated secondary antibody for ESM1, eEF2K, p-eEF2K and β-actin primary antibodies. Goat Anti-Mouse IgG H&L (HRP) (Cat. # ab205719, Abcam Plc, Cambridge, UK) was used as HRP conjugated secondary antibody for COL18A1 primary antibody.

Western blot technique was carried out with Mini Trans-Blot and Trans-Blot Turbo Transfer Systems (Bio-Rad Laboratories Inc., Hercules, California, USA). The membranes were incubated with Pierce ECL (Enhanced chemiluminescence) Western Blotting Substrate (Cat. # 32209, Thermo Fisher Scientific Inc., Waltham, Massachusetts, USA) and visualized by Kodak Gel Logic 100 Digital Imaging System (Eastman Kodak Company, Rochester, N.Y., USA). Finally, densitometry analyses of the protein bands were performed with Fiji (ImageJ) image processing software.

### Adipokine array

Freshly resected liver tissue samples were lysed in RIPA lysis and extraction buffer (Cat. # 89900, Thermo Fisher Scientific, Waltham, MA, USA) supplemented with Halt protease and phosphatase inhibitor cocktail (Cat. # 78442, Thermo Fisher Scientific, Waltham, MA, USA) for 30 min. Protein expression levels were investigated in remnant liver tissues obtained from the animals that were sacrificed 0 and 5 days after PH (3 samples from each group were pooled together) via a commercially available Proteome Profiler Rat Adipokine Array Kit (Cat. # ARY016; R&D Systems, Inc., Minneapolis, MN, USA), which simultaneously determines the relative expression levels of 30 different obesity-related molecules (including RANTES), according to the manufacturer's instructions (DePeralta et al. 2016).

### Determination of gene expressions by real-time QPCR

RNA isolations from 20 to 30 mg liver tissues were performed according to the RNeasy Mini Kit protocol (Qiagen, Hilden, Germany). RNA concentrations were determined by NanoDrop 1000 Spectrophotometer (Thermo Fisher Scientific Inc., Waltham, Massachusetts, USA). Complementary DNA was synthesized with Transcriptor High Fidelity cDNA Synthesis Kit (Roche, Basel, Switzerland) according to the manufacturer’s instructions. Real-time qPCR was performed using QuantiTect SYBR Green PCR Kit (Qiagen, Hilden, Germany). The qPCR data were analyzed using the Livak model (2^−ΔΔCt^). *Vegf* (VEGF_reverse 5′-GCTGGCTTTGGTGAGGTTTG-3′, VEGF_forward 5′-CGACAGAAGGGGAGCAGAAA-3′), *Pcna* (PCNA_reverse 5′-ACAGTGGAGTGGCTTTTGTGA-3′, PCNA_forward 5′-AAGTTTTCTGCGAGTGGGGA-3′) gene expressions were evaluated. *β-actin* (β-actin _reverse 5′-TATCCTGGCCTCACTGTCCA-3′, β-actin_forward 5′-AAGGGTGTAAAACGCAGCTCA-3′) was used as the reference gene.

### Enzyme-linked immunosorbent assay (ELISA)

Endocan and endostatin levels in serum samples taken from the rats on days 0, 1, 2, 3, 5, 7, 10, 14 after PH were determined by ELISA (Rat Endothelial Cell-Specific Molecule 1 / Endocan (ESM1) ELISA Kit, Cat. # abx256899, Abbexa Ltd., Cambridge, UK; Rat Endostatin ELISA Kit, Cat. # abx256898, Abbexa Ltd., Cambridge, UK) according to the manufacturer’s instructions.

### In silico analyses

In order to test and develop our hypothesis, we first used in silico methods on publicly available gene expression databases, since a key starting point in studying molecular mechanisms involves deciding how to identify critical biomolecules that will be investigated in in vivo experiments (Ekins et al. 2007). Microarray data analyses were performed using GSE4528, GSE55434, GSE70593 and GSE110292 microarray datasets by GEO2R web-based analysis tool (Davis and Meltzer 2007). GEO2R is an interactive tool that allows for comparing groups of samples in a GEO Series in order to determine genes which are differentially expressed across experimental conditions. Indeed, GEO2R performs comparisons on original data using the GEOquery and limma R packages from the Bioconductor project, which is an open source software project based on the R programming language that provides tools for the analysis of high-throughput genomic data (Smyth 2004; Smyth et al. 2005). The utilization of in silico strategies provides a neat framework for the initial identification of critical biomolecules (Murray et al. 2007). Indeed, online open-source gene expression data depicts a valuable resource for identifying differential gene expression. These datasets are collected and annotated in highly organized online databases (Murray et al. 2007). NCBI Gene Expression Omnibus (GEO) is such an international public repository that archives and freely distributes high-throughput functional genomics data such as microarray and next-generation sequencing (Barrett et al. 2013). Such resources are usually utilized as starting points in many research studies for the discovery of biomolecules.

### Statistical analyses

Data are presented as mean ± SD or median and interquartile range (25th–75th percentiles). After controlling for the parametric distribution assumptions, Wilcoxon test and Kruskal–Wallis test were used to examine differences among groups. Student’s t-test and Mann–Whitney U test were used for pairwise comparisons. Bonferroni correction was utilized for multiple pairwise comparisons to avoid the inflation of statistical significance. A 5% type-I error level was used to infer statistical significance. All statistical analyses were carried out using IBM SPSS Statistics for Windows Software version 23.

### Data availability

All data generated or analyzed during this study are included in this published article.

## Results

### In silico analyses revealed critical molecules can be implicated as part of a hepatostat in liver regeneration

**Fig. 2:**
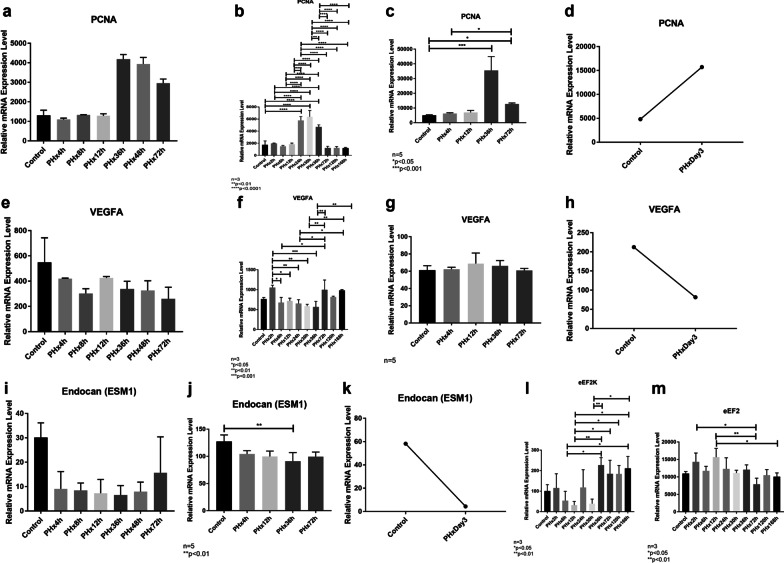
*Pcna* results of in silico analyses of GSE4528 (**a**), GSE55434 (**b**), GSE70593 (**c**) and GSE110292 (**d**) microarray datasets utilizing GEO2R. *Vegf* results of in silico analyses of GSE4528 (**e**), GSE55434 (**f**), GSE70593 (**g**) and GSE110292 (**h**) microarray datasets utilizing GEO2R. Endocan results of in silico analyses of GSE4528 (**i**), GSE70593 (**j**) and GSE110292 (**k**) microarray datasets utilizing GEO2R. eEF2K (**l**) and eEF2 (**m**) results of in silico analyses of GSE55434 microarray dataset utilizing GEO2R

In terms of in silico investigations, we used 4 different datasets in the GEO database. We performed in silico data analyses using GSE4528, GSE55434, GSE70593 and GSE110292 microarray datasets from GEO. Figure 2 demonstrates microarray results from each of these datasets. Microarray data analyses demonstrated that *Pcna* expression increased 24 h hour after PH, and peaked at 36 h (Fig. 2a [GSE4528] and Fig. 2c [GSE70593]). Moreover, *Pcna* expression decreased on the advancing hours (Fig. 2). Lastly, it was observed that *Pcna* expression increased 72 h after PH compared to the control (Fig. 2d [GSE110292]). All these meta-analysis results are consistent with the data we have obtained (vide infra).

On the other hand, we found that *Vegf* showed a decreasing trend until day-3 after PH (Fig. 2e [GSE4528]). *Vegf* was shown to increase 2 h after PH, but it decreased on the advancing hours compared to the control (Fig. 2f [GSE55434]). Similarly, it was observed that *Vegf* expression decreased 72 h after PH (Fig. 2h [GSE110292]). In addition, *Vegf* expression was shown not to change (Fig. 2g [GSE70593]). Furthermore, our in silico analyses demonstrated that endocan tended to decrease over time after PH (Fig. 2i–k).

Last but not least, the in silico analyses demonstrated a decrease in eEF2K transcription level in the early hours after PH, whereas an increase was observed on the advancing hours (Fig. 2l [GSE55434]). Therefore, it can be deduced that overall protein translation seems to be regulated on the advancing hours. The levels of eEF2 (Fig. 2m) seem to be inversely correlated with eEF2K (Fig. 2l), as expected.

Although in silico analyses represent an excellent resource as a starting point for biomolecule discovery, experimental validation of in silico derived results is needed (Murray et al. 2007). As such, we aimed to reveal mechanistic insights into liver regeneration by examining PCNA, VEGF, endocan, endostatin and eEF2K levels after PH via utilizing an in vivo liver regeneration model in rats (vide infra).

### Partial hepatectomy induces robust liver regeneration

**Fig. 3:**
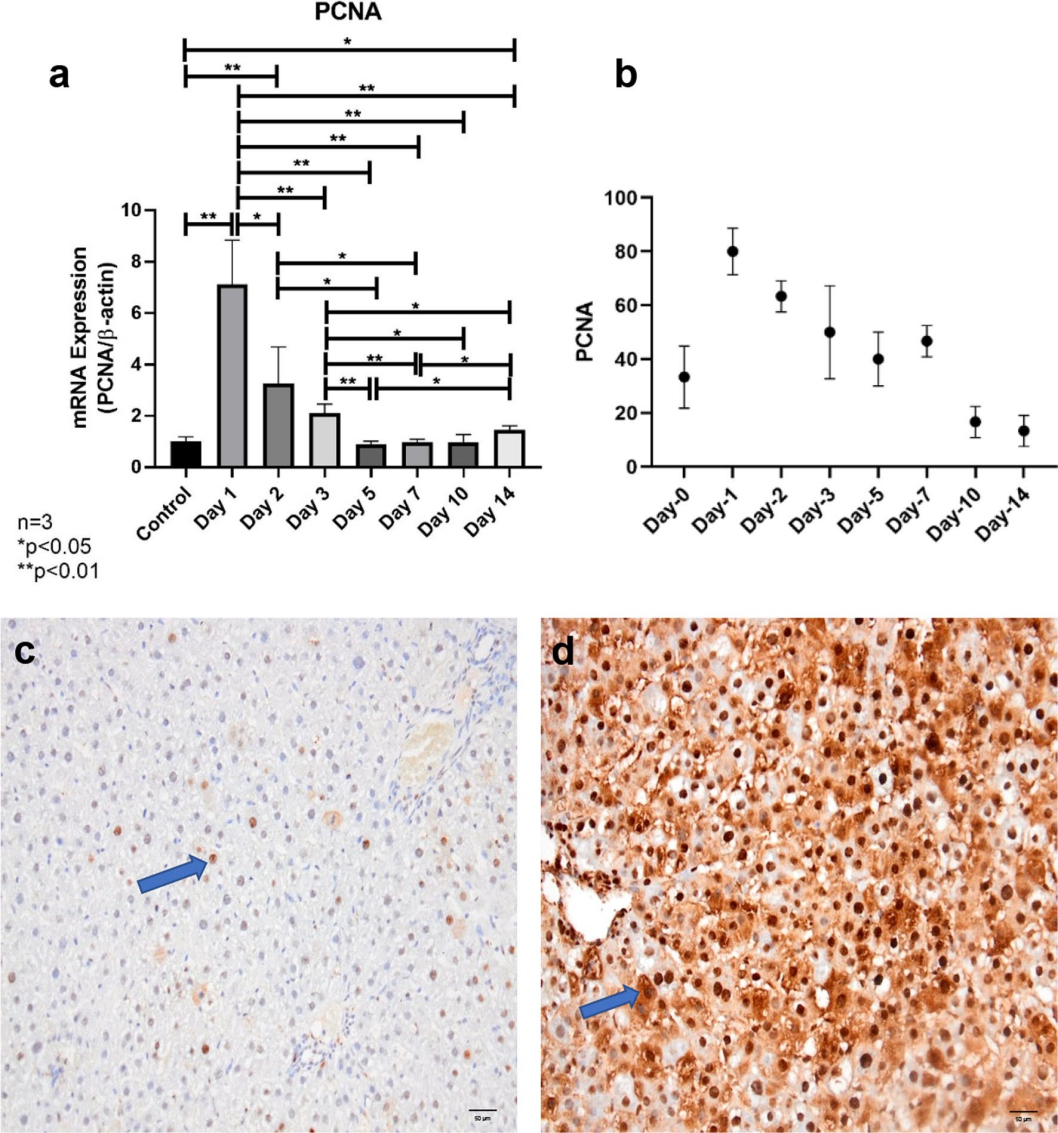
**a** Induction of liver regeneration after partial hepatectomy revealed by RT-qPCR analyses. *Pcna* mRNA expression levels of liver tissues obtained from the rats demonstrated that highest proliferative activity was observed on day-1. *Pcna* expression gradually declined after 48 h and returned to its baseline value on day-5 after partial hepatectomy. **b** Induction of liver regeneration after partial hepatectomy revealed by immunohistochemistry. The PCNA labeling index was calculated as the proportion of PCNA-positive hepatocytes. Immunohistochemical evaluations of PCNA expressions of liver tissues obtained from the rats demonstrated that highest proliferative activity was observed on Day-1. PCNA expression gradually declined after 48 h and returned to its baseline value on Day-5. **c** Representative image of immunohistochemical staining showing hepatocyte nuclei stained with PCNA on Day-0 (control). PCNA proliferative activity of hepatocytes was determined as approximately 10% (× 200) (scale bar, 50 μm). **d** Representative image of immunohistochemical staining showing hepatocyte nuclei stained with PCNA in residual liver tissue on Day-1 after partial hepatectomy. Most of the hepatocytes (90%) expressed PCNA (× 200) (scale bar, 50 μm). (PCNA-positive nuclei are marked with blue arrows, indicating the cells in S-phase of the cell cycle)

Real-time qPCR analyses of *Pcna* mRNA expression in tissue samples revealed that *Pcna* was not at all expressed in rat liver tissues obtained during the initial operation (Fig. 3a). Our data demonstrated that PCNA expression level in residual liver tissues peaked 24 h after PH (p < 0.01). Nuclear PCNA expression also confirmed these findings (Fig. 3b–d). Our results concerning *Pcna* expression demonstrated that highest proliferative activity was observed on day-1. *Pcna* expression gradually declined after 48 h and returned to its baseline value on day-5.

### Partial hepatectomy induces angiogenesis

**Fig. 4:**
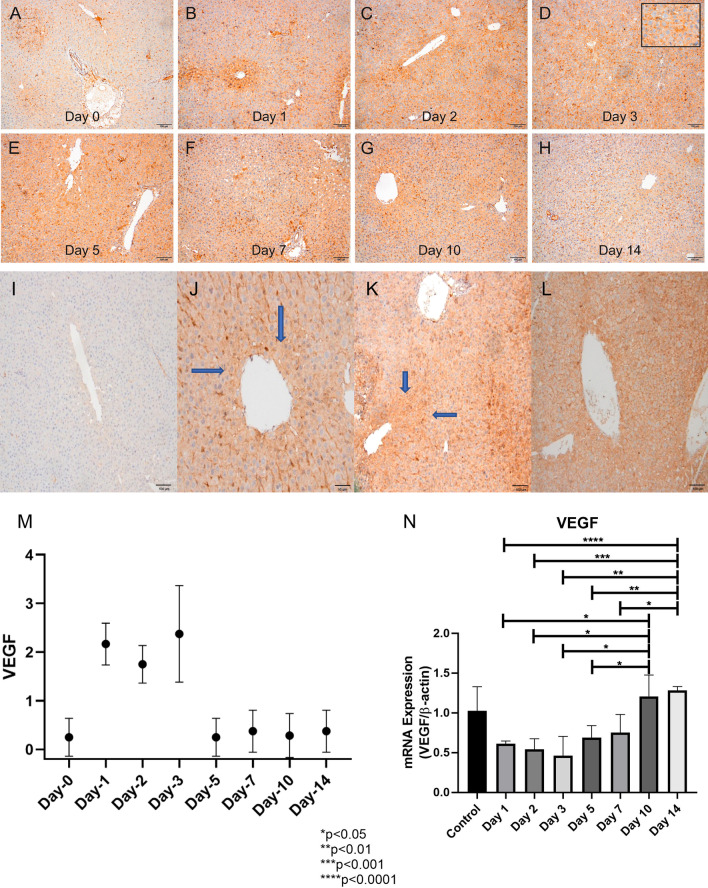
Representative images of immunohistochemical stainings showing liver tissues stained with VEGF on day-0 (control) (**a**), − 1 (**b**), − 2 (**c**), − 3 (**d**), − 5 (**e**), − 7 (**f**), − 10 (**g**) and − 14 (**h**) (× 100) (scale bar, 100 μm). **i**–**l** Staining patterns of hepatocytes with VEGF, gradually progressing from the periportal area to the pericentral area. **i** Score 0, no staining (× 100) (scale bar, 100 μm). **j** Score 1, expression limited to periportal hepatocytes (× 200) (scale bar, 50 μm). **k** Score 2, VEGF expression in periportal and perisinusoidal hepatocytes (× 100) (scale bar, 100 μm). **l** Score 3, complete VEGF staining in all hepatocytes including pericentral areas (× 100) (scale bar, 100 μm). Blue arrows indicate VEGF positive hepatocytes. **m** Induction of angiogenesis after partial hepatectomy revealed by immunohistochemistry. The level of VEGF expression was evaluated based on a four-tiered scoring system on a scale ranging from 0 to 3 (as explained in the methods section [vide supra]). Immunohistochemical evaluations of VEGF expressions of liver tissues obtained from the rats demonstrated that highest angiogenesis activity was observed on days 1–3. VEGF expression gradually declined after 72 h and returned to its baseline value on day-5. **n** Induction of liver angiogenesis after partial hepatectomy. *Vegf* mRNA expression levels of liver tissues obtained from the rats demonstrated that highest angiogenesis activity was observed on day-14. *Vegf* expression gradually declined on days 1–3 and started to increase after day-3

Immunohistochemcial assessments of VEGF expression in liver tissue samples revealed that VEGF was not at all expressed in rat liver tissues obtained during the initial operation (Fig. 4i–l). Our data demonstrated that VEGF expression level in residual liver tissues peaked 24–72 h after PH. Indeed, the first significant increase in VEGF immune reactivity in hepatocytes was on day-1. VEGF expression levels were found to be upregulated also on day-2 and day-3. VEGF immunoreactivity started to decline as of day-5 and returned to its basal level (Fig. 4i–m).

On the other hand, real-time qPCR analyses of *Vegf* mRNA expression in liver tissue samples revealed that whole tissue expression of *Vegf* mRNA demonstrated a different kinetic compared to immunohistochemical analyses (Fig. 4n). The average level of *Vegf* mRNA expression of the liver tissue seemed to increase after day-5 and reached its highest level on day-14.

### Partial hepatectomy induces a decrease in liver tissue endocan levels

**Fig. 5:**
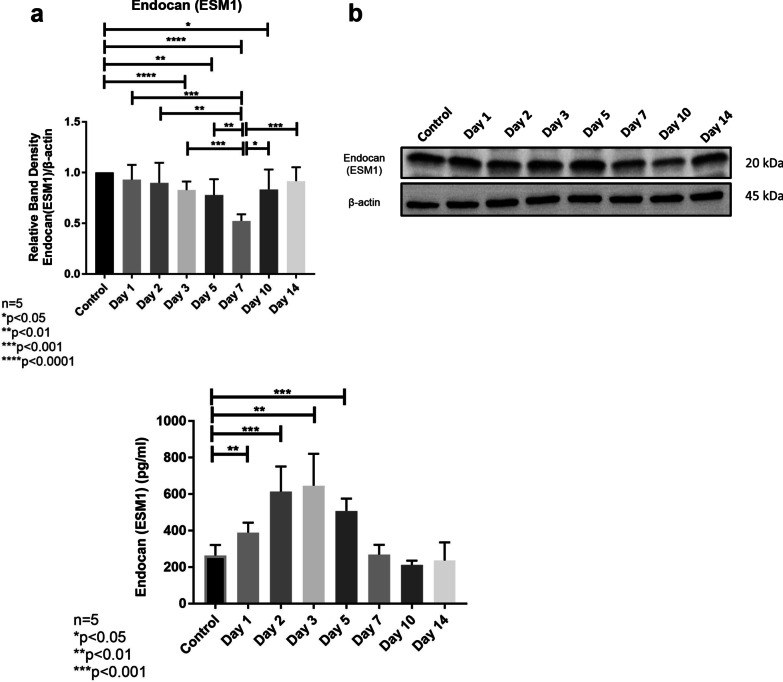
**a**, **b** Endocan protein levels in remnant liver tissue samples after partial hepatectomy revealed by Western blot analyses. Endocan levels gradually decreased after partial hepatectomy, reaching the lowest level on day-7. **c** Serum endocan levels after partial hepatectomy revealed by ELISA analyses. Endocan levels gradually increased after partial hepatectomy and reached the highest level on day-3. Then, endocan levels gradually decreased and returned to basal level on day-7

Investigation of endocan protein levels in liver tissue samples after PH revealed that endocan activity seemed to decline gradually on days 1–7 compared to the control group (day-0). The first statistically significant decline in tissue endocan activity occurred on day-3 (p < 0.05). Tissue endocan level reached its lowest value on day-7 (p < 0.05) (Fig. 5a, b).

### The decrease in liver tissue endocan expression was accompanied by an increase in serum endocan levels

Investigation of serum endocan levels after PH demonstrated that the first significant increase in serum endocan activity was observed on day-1 (p < 0.01) (Fig. 5c). Serum endocan level reached its highest level on day-3. Furthermore, our analyses revealed that serum endocan levels gradually decreased after day-3. Although serum endocan level on day-5 was still significantly higher than that on day-0 (p < 0.01), it reached its basal level on day-7 (Fig. 5c).

### Partial hepatectomy induces a rapid increase in liver tissue endostatin levels

**Fig. 6:**
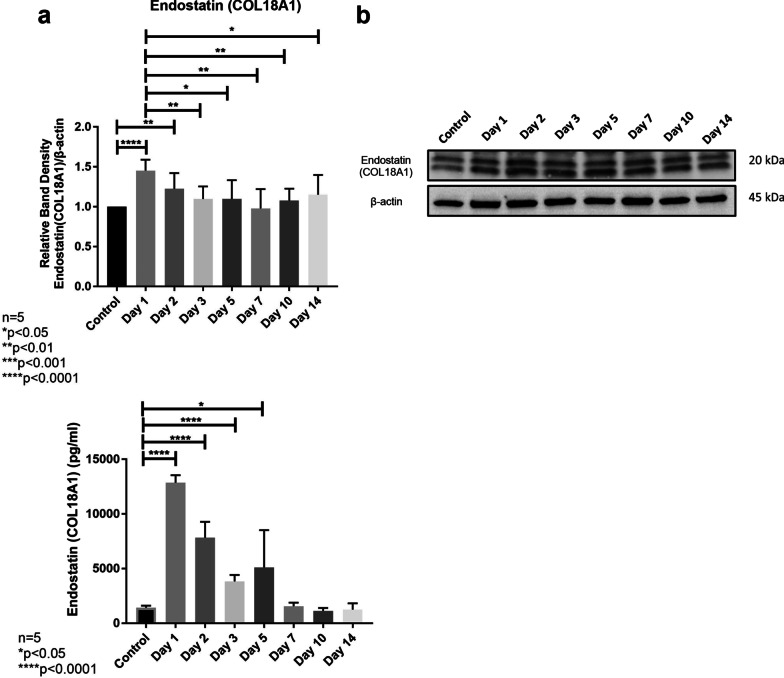
**a**, **b** Endostatin protein levels in remnant liver tissue samples after partial hepatectomy revealed by Western blot analyses. Endostatin is a 20 kDa C-terminal fragment of collagen XVIII. Endostatin levels peaked on day-1 after partial hepatectomy and then gradually declined, reaching the lowest level on day-7. **c** Serum endostatin levels after partial hepatectomy revealed by ELISA analyses. Endostatin levels rapidly increased after partial hepatectomy and reached the highest level on day-1. Then, levels of endostatin gradually decreased and returned to basal level on day-7

Investigation of endostatin protein levels in liver tissue samples after PH showed that endostatin expression rapidly and significantly increased on day-1 in comparison to Day-0 (p < 0.05), reaching its highest level. Following this surge, endostatin expression gradually declined starting from day-2 (Fig. 6a, b).

### The surge in liver tissue endostatin expression on day-1 was accompanied by a peak in serum endostatin level

We observed a significant increase in serum endostatin level on day-1 compared to day-0 (Fig. 6c). Indeed, this significant increase in serum endostatin activity on day-1 represented the peak value after PH (p < 0.0001). Serum endostatin levels gradually decreased after day-1; however, they stayed higher than day-0 until day-5. Serum endostatin then returned to basal level on day-7.

### Partial hepatectomy induces regulation of eEF2K as well as RANTES

**Fig. 7:**
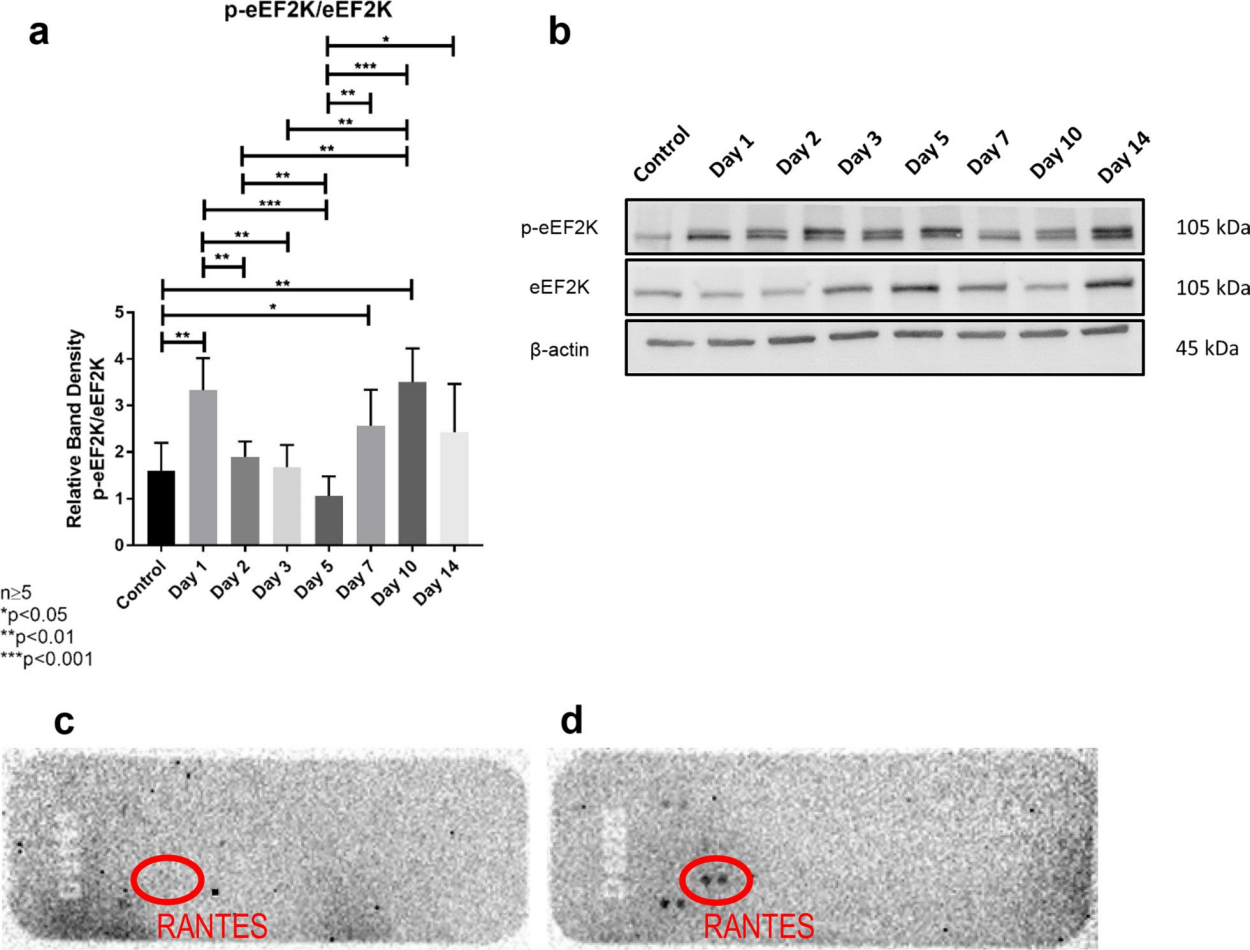
**a**, **b** p-eEF2K/eEF2K protein levels in remnant liver tissue samples after partial hepatectomy revealed by Western blot analyses. p-eEF2K levels peaked on day-1 after partial hepatectomy and then gradually declined, reaching the lowest level on day-5. Then, p-eEF2K seemed to increase on the advancing hours. **c**, **d** Results of adipokine array analyses in remnant liver tissue samples after partial hepatectomy. RANTES level increased on day-5 (**d**) in comparison to day-0 (**c**). Each visible spot on the array is a technical duplicate from a panel of adipokines. RANTES (identified by red circle) was identified to be up-regulated on day-5 in comparison to day-0

We also investigated the activation status of eEF2K by analyzing p-eEF2K and eEF2K protein expression levels by Western blot. eEF2K, which suppresses protein synthesis during elongation phase, phosphorylates eEF2 and inhibits its function (Liao et al. 2016). We demonstrated that the eEF2K inhibitory phosphorylation site peaked on the first day; thus, increasing protein translation. We demonstrated that protein translation is also regulated on the advancing hours (Fig. 7a, b). Moreover, we utilized a proteome profiler adipokine array, which simultaneously detects the relative levels of 30 different adipokine molecules in the samples investigated. RANTES (regulated on activation, normal T cell expressed and secreted [also known as CCL5]) (Madani et al. 2009; Krensky and Ahn 2007), which is a small CC chemokine that functions by binding to three receptors (CCR1, CCR3, and CCR5) (Berres et al. 2010), was identified from this assay to show greater protein expression on day-5 in comparison to day-0 (Fig. 7c, d). RANTES has been implicated in hepatic wound healing response (Berres et al. 2010; Affo and Bataller 2011). In addition, the CCL5/CCR5 axis was demonstrated to be an important system in the hepatic would healing process. Indeed, RANTES (CCL5) was previously found to be induced in murine and human liver after injury (Berres et al. 2010; Affo and Bataller 2011).

## Discussion

**Fig. 8:**
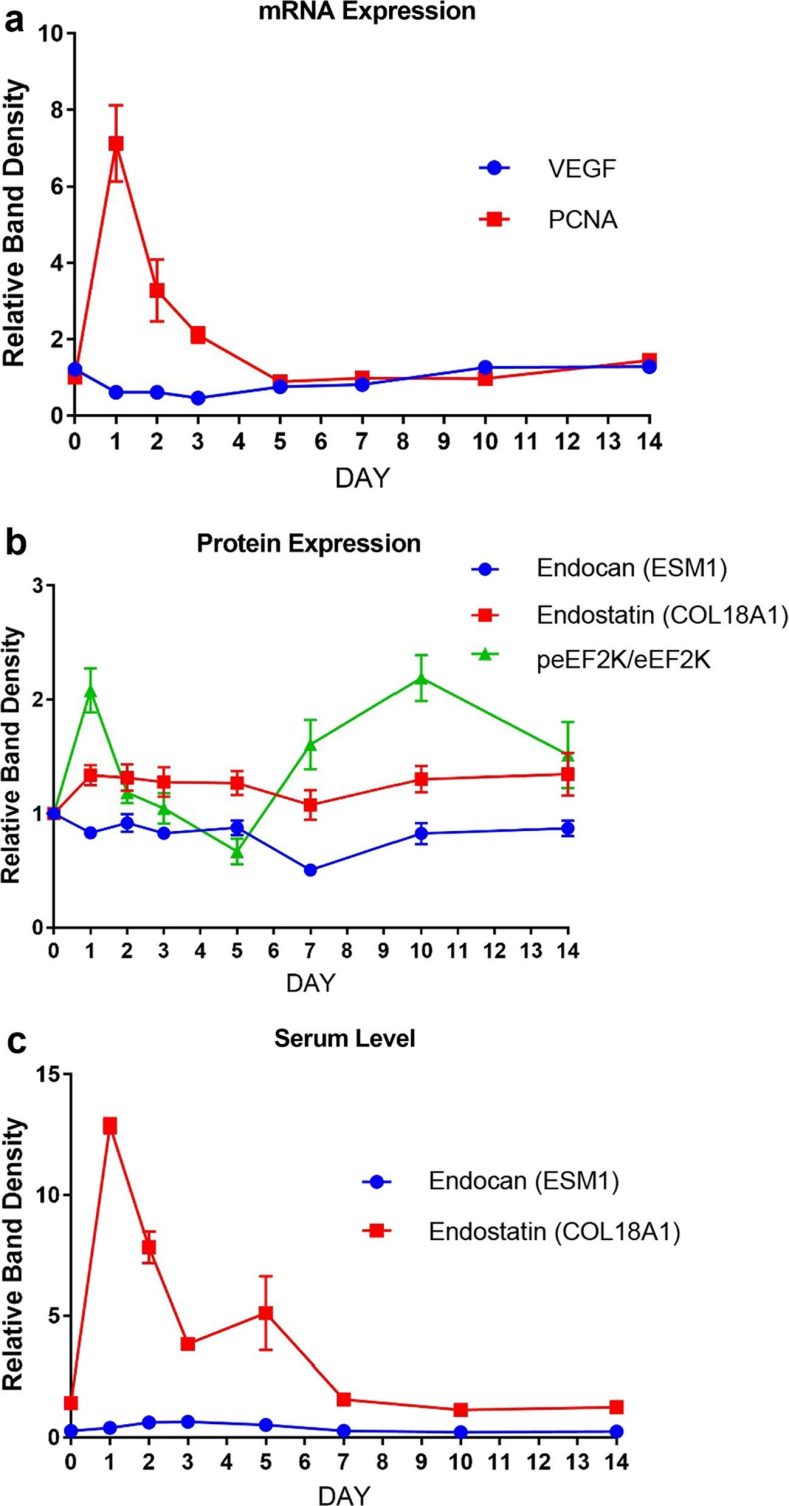
Tissue mRNA and protein expressions as well as serum levels of factors implicated in liver regeneration after partial hepatectomy. Temporal changes of tissue mRNA levels of *Vegf* and *Pcna* (**a**); tissue protein expression levels of endocan, endostatin, p-eEF2K/eEF2K (**b**); and serum levels of endocan and endostatin (**c**) after partial hepatectomy

The regeneration process in the remaining liver reaches the maximum level within 24-48 h (Dogrul et al. 2010). The rapid proliferative activity usually decreases by day-3 (Michalopoulos and DeFrances 1997). Previously, it was shown that the liver achieves its optimal function and mass on days 7–10 after PH in rats. In line with our findings, Zhao et al. showed that PCNA immune reactivity after PH increased significantly on days 1–3 (Zhao et al. 2020b), although several other studies reported a significant increase in PCNA immune reactivity between days 2–7 after PH, returning to baseline value on day-10 (Dogrul et al. 2010). Our findings clearly revealed that the proliferative activity appears to decline gradually after day-1, returning to basal level on day-5 (Fig. 8a).On the other hand, *Vegf* mRNA expression in liver tissue samples revealed that whole tissue expression of *Vegf* mRNA demonstrated a different kinetic compared to immunohistochemical analyses. We postulated that the difference in *Vegf* mRNA expression kinetic might be due to the fact that the analyses were performed on whole tissue lysates. Therefore, average mRNA expression levels of the liver parenchyma as a whole might indeed be very different from the expression of VEGF on endothelial cells, which may be displayed by immunohistochemical assessments. It was shown that angiogenesis first begins in the periportal area and then progresses to the pericentral area. Our findings demonstrated that angiogenesis after PH starts from the periportal area, where hepatocytes in the regeneration process first start to regenerate. In light of our findings, we contemplate that VEGF expression and regeneration peaked on day-3. The initiation of angiogenesis from the periportal area, where the hepatocytes start to regenerate, supports the fact that regeneration and angiogenesis act synergistically. The lack of statistical significance in VEGF expression after 72 h might suggest that angiogenesis-independent factors may also play critical roles in terms of achieving the optimal mass of the liver after PH. In a previous seminal study of our group, we demonstrated that robust angiogenesis started on day-3 and returned to the baseline value on day-5 (Dogrul et al. 2010). Dink et al. showed that the expression of VEGF in the liver starts to increase 26 h after the PH and reaches the peak value at 72 h (Ding et al. 2010). Similarly, Shimizu et al. demonstrated that VEGF expression peaked 72 h after PH and then declined (Shimizu et al. 2005). Yoshida et al. also reported similar results (Yoshida et al. 2011). Moreover, inhibition of VEGF activity has also been shown to significantly inhibit the proliferative activity of hepatocytes 48 h and 96 h after PH (Taniguchi et al. 2001).

Previous studies by our group demonstrated that endostatin produced by hepatocytes might modulate hepatic regeneration by inhibiting angiogenesis (Dogrul et al. 2010). A significant relationship between post-hepatectomy regeneration capacity and serum endostatin level was previously demonstrated for normal liver (Dogrul et al. 2010; Colakoglu et al. 2007). In light of our findings, the expression of endostatin in liver tissue reached its peak value on day-1, which is indeed parallel with the serum levels of endostatin (Fig. 7c, d). The simultaneous increases of the serum endostatin levels and VEGF expression in the remnant liver tissues suggest that proangiogenic and antiangiogenic factors might be functioning in a dynamic reciprocal balance during the systemic response of liver regeneration.

Our findings demonstrated that p-eEF2K levels showed two peaks, *i.e.*, on day-1 and day-10 (Fig. 8b). Thus, protein translation seems to be maximally upregulated on these days. Given our findings showing that liver regeneration reaches its peak on day-1 (Fig. 8a) as well as the significant increases of VEGF expression in hepatocytes, serum endocan and endostatin levels at 24 h (Fig. 8c), the first peak of eEF2K on day-1 (Fig. 8b) suggests that it may assume pivotal roles together with other factors during liver regeneration. In addition, Zhou et al. recently reported that eEF2K also took active part in angiogenesis via SP1/KLF5‐mediated VEGF expression, as well as the subsequent stimulation of PI3K/Akt and STAT3 signaling (Fig. 1 depicts the summary of critical findings from the literature as well as proposing a probable role for endocan in hepatic regeneration) (Zhou et al. 2020). Moreover, the role of eEF2K is not limited to this initial response, as its levels also significantly increase on the advancing hours. In addition, we also found that protein expression level of RANTES, which has been implicated in liver recovery and regeneration, increased 5 days after PH (Chen et al. 2020; Li et al. 2020).

In healthy tissues, endocan is expressed by endothelial cells (*e.g.* lung, kidney). Endocan regulation is impaired in several diseases including cancer (Iozzo and Cohen 1993). Li et al. proposed that endocan may show the degree of endothelial cell injury in renal allografts (Li et al. 2012). However, no study has evaluated the role of endocan in terms of hepatic regeneration. Therefore, we then set out to explore the role of endocan during liver regeneration after PH. The first significant increase in serum endocan level was observed on day-1, while it reached its highest level on day-3. Despite the significant increase of the serum endocan levels until day-5, its levels tended to decline after day-5. The serum endocan levels returned to basal level on day-7. Since the temporal kinetic of serum endocan levels resembled that of VEGF, we think serum endocan may indeed be implicated in angiogenic response as a molecular “*hepatostat*”. Expressions of angiogenic growth factors were shown to be associated with the expression of endocan (Gerritsen et al. 2003). Direct interaction between these angiogenic growth factors and endocan seems to result in the regulation of angiogenesis. Thus, it should come as no surprise that endocan blockade suppressed neovascularization (Su et al. 2018).There exist various studies in the literature suggesting that liver regeneration is an angiogenesis-dependent process. Moreover, angiogenesis was reported to be associated with endocan in most tumors (Chen et al. 2010). However, no study has investigated the role of endocan in angiogenesis and regeneration after PH, yet, to the best of our knowledge. Therefore, this is the first study to reveal the associations of serum and tissue endocan levels with liver regeneration after PH. Our results suggest that serum endocan values peaked simultaneously with angiogenic response. Furthermore, we also observed that serum endocan level reaches the lowest value on day-10 and day-14, which represent the time-point of reaching ideal liver mass/volume. Thus, we propose that the serum endocan level might prove to be an important biomarker in liver regeneration with further studies. Indeed, tissue endocan levels decline gradually on days 1–7, in contrast to the control group, reaching the lowest value on day-7. Interestingly, the gradual decrease of tissue endocan during days 1–7 and the dip on day-7 closely resemble the changes observed in terms of tissue endostatin levels (Fig. 8b). Indeed, we also observed the lowest tissue levels of endostatin on day-7. Since the temporal profiles of tissue endocan and endostatin levels are similar (Fig. 8b), our data suggest that remnant liver tissue endostatin and endocan may act synergistically after PH.

Endocan might play important roles in regulation of the normal cellular processes such as proliferation, remodeling, migration or angiogenesis. It can be considered as an accurate marker of endothelial activation (Delehedde et al. 2013). In addition, endocan was shown to bind and activate hepatocyte growth factor (HGF), (Bechard et al. 2001b) which is a major hepatocyte mitogen (Bohm et al. 2010). Serum levels of HGF significantly increase within 1–3 h after PH (Michalopoulos 2007). VEGF-A, which is known to be crucial for blood vessel formation, is also strongly upregulated in the regenerating liver, especially in hepatocytes (Taniguchi et al. 2001). VEGF-A mediated activation of VEGFR-1 results in the release of HGF from endothelial cells (Fig. 1) (LeCouter et al. 2003). Intriguingly, LeCouter et al. reported that VEGF-A did not stimulate growth of hepatocytes in vitro, unless endothelial cells were also present in the culture. HGF was proposed be an endothelial cell-derived paracrine mediator which promoted hepatocyte growth (Fig. 1) (LeCouter et al. 2003). Furthermore, endocan expression was shown to be stimulated by VEGF-A through the phosphorylation and activation of VEGFR-2, which was required to promote cell migration and tube formation by VEGF-A (Roudnicky et al. 2013). VEGF-mediated induction of endocan was shown to be positively and negatively regulated by PKC/NFκB and PI3 K/AKT/FKHRL1 signaling pathways, respectively (Delehedde et al. 2013). These findings clearly suggest a pivotal role for endocan in terms of liver regeneration. In addition to VEGF-A, various molecules have been implicated in the regulation of endocan expression. Tumor necrosis factor-α (TNF-α), interleukin-1 (IL-1), TGF-β1, fibroblast growth factor-2 and (FGF-2) have been demonstrated to induce endocan expression (Fig. 1) (Gerritsen et al. 2003; Dieterich et al. 2012; Maurage et al. 2009; Kirwan et al. 2005; Rennel et al. 2007; Zhao et al. 2004). Terán et al. demonstrated that eEF2 controlled TNF-α translation. Inhibitory phosphorylation of eEF2K promoted eEF2 activation and subsequent TNF-α elongation (Gonzalez-Teran et al. 2013). Such findings proposed a new signaling pathway that regulated TNF-α production in liver damage. Indeed, our finding of the peak of eEF2K on day-1 (Fig. 8b) may be critical in terms of regulating endocan activity as well liver regeneration via TNF-α. On the other hand, interferon-γ (IFN-γ) inhibits TNF-α induced upregulation of endocan (Lassalle et al. 1996). Expressions of TNF-α and IL-6 are induced in Kupffer cells after PH. In addition to its effect on endocan, TNF-α further enhances the expression of IL-6 (Fig. 1) (Bohm et al. 2010). IL-6 signals via the IL-6 receptor expressed by hepatocytes. This results in activation of STAT3 which induces hepatocyte proliferation after PH (Fig. 1) (Bohm et al. 2010).

## Conclusion

In the current study, we demonstrated the association of endocan with other molecules implicated in liver regeneration and angiogenesis after PH. Thus, modalities targeting endocan activity may offer novel rational strategies to facilitate or control liver regeneration after various conditions including living liver donation or xenobiotic-induced liver injury. Moreover, endocan might as well serve as a useful biomarker to monitor clinical prognosis in a plethora of conditions such as the recovery of donor's remaining liver after living-donor liver transplant. Whether endocan might represent a strategy to optimize liver regeneration when given therapeutically needs to be investigated in future studies. Since a limitation of the current study was the absence of functional analyses regarding the blocking of endocan, we plan to investigate the magnitude of the effects of endocan on liver regeneration via experiments that utilize antagonists or endocan-*knock-out* animals.

In conclusion, we utilized a surgically induced rat PH model to investigate the probable role of endocan in liver regeneration and to reveal its temporal kinetic interactions with other endogenous molecules. In future studies, we plan to further clarify the intracellular effects of endocan in terms of liver regeneration and angiogenesis as well as regulation of neovascularization. We think the findings of this study may provide support for other studies that investigate the critical mechanisms underlying liver regeneration.

## Data Availability

All data generated or analyzed during this study are included in this published article.
